# Grain yield of common bean (*Phaseolus vulgaris* L.) varieties is markedly increased by rhizobial inoculation and phosphorus application in Ethiopia

**DOI:** 10.1007/s13199-017-0529-9

**Published:** 2017-12-19

**Authors:** Tarekegn Yoseph Samago, Endalkachew W. Anniye, Felix D. Dakora

**Affiliations:** 1 Department of Crop Sciences, Tshwane University of Technology, Pretoria, 0001 South Africa; 2 School of Plant and Horticultural Sciences, Hawassa University, Awasa, Ethiopia; 3 Department of Chemistry, Tshwane University of Technology, Private Bag X680, Pretoria, 0001 South Africa; 4 Present Address: Department of Chemistry, Tshwane University of Technology, Private Bag X680, 175 Nelson Mandela Drive, Pretoria, 0001 South Africa

**Keywords:** δ^15^N, %Ndfa, N contribution, Reference plants, *Rhizobium* strain

## Abstract

A field experiment was conducted to assess plant growth, symbiotic performance and grain yield of common bean in response to rhizobial incoculation and phosphorus application at Galalicha in Southern Ethiopia during the 2012 and 2013 cropping seasons under rain-fed conditions. The treatments consisted of 2 released common bean varieties (Hawassa Dume and Ibbado), 3 levels of *Rhizobium* inoculation (uninoculated, inoculated with strain HB-429 or GT-9) and 4 levels of phosphorus application (0, 10, 20 and 30 kg P ha^−1^) using a split-split plot design with four replications. Here, phosphorus levels, *Rhizobium* inoculation and common bean varieties were assigned as main, sub- and sub-sub treatments, respectively. The results revealed marked varietal differences in plant growth, grain yield and symbiotic performance. Of the two common bean varieties studied, Hawassa Dume generally showed superior performance in most measured parameters in 2013. *Rhizobium* inoculation significantly (*p* ≤ 0.05) increased plant growth, symbiotic performance and grain yield. Applying *Rhizobium* strain HB-429 to bean crop respectively increased plant growth, %Ndfa, amount of N-fixed and grain yield by 19, 17, 54 and 48% over uninoculated control. Similarly, the application of 20 kg P ha^−1^ to bean plants respectively resulted in 36, 20, 96 and 143% increase in plant growth, %Ndfa, N-fixed and grain yield when compared to the control. These results clearly indicate that plant growth, symbiotic performance and grain yield of common bean can be significantly increased by *Rhizobium* inoculation and phosphorus fertilization in Ethiopia. *Rhizobium* inoculants are a cheaper source of nitrogen than chemical fertilizers and when combined with moderate phosphorus application can markedly increase grain yield for resource-poor farmers.

## Introduction

Organic and chemical fertilisers are the two main agricultural inputs applied to crops by large scale farmers in Africa. These fertilisers are however too expensive for resource-poor smallholder farmers in the continent (Ngetich et al. 2012; Fukuda et al. 2012). N_2_-fixing legumes are a cheaper and more sustainable alternative to chemical N fertilizers for small-scale farmers in Africa. Nodulated legumes are known to contribute substantial amounts of symbiotic N to cropping systems (Belane and Dakora 2009, 2010; Naab et al. 2009; Nyemba and Dakora 2010; Sprent et al. 2010; Pule-Meulenberg et al. 2010; Mohale et al. 2014). The inclusion of nodulated grain legumes like common bean in cropping systems can improve crop yields and thus replenish soil N (Maina et al. 2011).

N_2_ fixation in legumes is the second most important biological process after photosynthesis (Hayat et al. 2008; Unkovich et al. 2008), and contributes N to meet the legume’s N demand, as well as for succeeding crops (Peoples et al. 2009). Rhizobial strains including *Rhizobium leguminosarum* biovar *phaseoli* are therefore widely used as inoculants to improve growth, symbiotic performance and grain yield of common bean under both glasshouse and field conditions (Kellman et al. 2005). However, N_2_ fixation by the common bean symbiosis with soil rhizobia is hardly adequate to meet plant growth and grain yield (Kabahuma 2013; Sánchez et al. 2014). This is due, in part, to susceptibility of the crop to nutritional and environmental constraints, its short maturity period, and the ineffectiveness of indigenous soil rhizobia (Kabahuma 2013). Pre-inoculation of seeds with elite rhizobial strains was shown to increase symbiotic performance and grain yield of common bean planted in farmers’ fields (Akter et al. 2014; Tabaro 2014). Percent N derived from fixation of atmospheric N_2_ and N contribution have been estimated from the δ^15^N values of *Rhizobium*-inoculated common bean (Bambara and Ndakidemi 2010a, b), and from farmers’ fields where no inoculants were applied (Nyemba and Dakora 2010).

Globally, low availabile P in many weathered tropical soils is a major constraint to common bean production (Lynch et al. 2009), as the P requirement of legumes is greater when compared to cereals (Silva et al. 2014). In Africa, the low P availability is exacerbated by soil degradation and inadequate fertilisation especially in smallholder agriculture, even though P supply to common bean has been shown to enhance nodulation, symbiotic efficiency and the uptake of mineral nutrients (Stamford et al. 2007; Mandri et al. 2012; Neila et al. 2014; Tajini and Drevon 2014). Furthermore, *Rhizobium* inoculation when combined with P fertilisation has been found to increase nitrogenase activity, plant growth, grain yield and soil fertility (Fatima et al. 2007; Hayat et al. 2008). In fact, combining rhizobial inoculation with P fertilisation is known to further enhance symbiotic N_2_ fixation and grain yield in nodulated legumes (Zafar-Allah et al. 2007; Bhuiyan et al. 2008; Bekere and Hailemariam 2012; Mfilinge et al. 2014).

P fertilisation of common bean in the field has also led to increased shoot biomass and root nodulation (Mourice and Tryphone 2012; Namugwanya et al. 2014). So far, however, there are no reports of bean response to rhizobial inoculation in combination with P fertilisation in Ethiopia. Therefore, the aim of this study was to assess the effect of *Rhizobium* inoculation and P application on plant growth, symbiotic performance and grain yield of two common bean varieties grown at Galalicha in Southern Ethiopia.

## Materials and methods

### Site description

A field experiment was conducted at Galalicha in Southern Ethiopia during the main rainy season (August to November) of 2012 and 2013. The site is located in South West of Hawassa town between 06^0^ 58^′^ - 07^0^ 02^′^ N latitude and 038^0^ 18^′^ - 038^0^ 19^′^ E longitude at an altitude of 1740–2000 m above sea level. The area respectively received a total rainfall of 744.1 and 756.8 mm during the 2012 and 2013 cropping seasons. Based on national metreological data, the average yearly minimum and maximum temperatures were 14.2 and 27.0 °C in 2012 and 2013, respectively. The soil at study site was a sandy-loam with pH 7.0 and 6.4, organic carbon 3.3 and 4.1 mg.g^−1^, total N 0.1 and 0.6 mg.g^−1^, plant-available P 0.65 and 1.00 mg.g^−1^, CEC 9.6 and 22.2 cmol.kg^−1^, K 2.2 and 3.1 cmol.kg^−1^, Ca 4.3 and 4.4 cmol.kg^−1^ and Mg 0.8 and 0.9 cmol.kg^−1^ in 2012 and 2013, respectively. In general, the soil analysis results indicated that the area is nutrient deficient to support the potential crop production.

### Source of planting material

Seeds of *Phaseolus vulgaris* L. varieties Hawassa Dume and Ibbado were obtained from the Hawassa Agricultural Research Centre, Hawassa, Ethiopia. The two common bean varieties were chosen based on their high grain yield, acceptability by farmers and seed availability. Hawassa Dume grows as an indeterminate semi-bush, takes about 85–110 days to physiological maturity, and has a dark-red seed coat pigmentation. In contrast, Ibbado grows as a determinate bush, takes 90–95 days to mature and has a speckled-red seed coat colour.

### Experimental design and treatments

Seeds were planted in a split-split-plot design which had a total of 24 treatment combinations with four replicate plots for each treatment. The field plot treatments included: i) addition of 10, 20 and 30 kg P ha^−1^ to soils as triple super phosphate (TSP) by banding along planting rows to a depth of 15 cm to avoid direct contact with seeds at the time of planting, and ii) *Rhizobium* inoculation of 1 kg seed with a peat based 10 g inoculant (Rice et al. 2001) containing 6.5 × 10^8^ viable bacterial cells g^−1^ peat of *Rhizobium leguminosarum* biovar *phaseoli* strain HB-429 (National Soil Research Laboratories, Microbiology Unit, Addis Ababa, Ethiopia), or *phaseoli* R.l.bv. strain GT-9 (Soygro Pty Ltd., Potchefstroom, South Africa). These two rhizobial strains are used as commercial inoculants for common bean cultivation in Ethiopia and South Africa. Uninoculated plants and plants grown in zero-P soils were included as controls.

### *Rhizobium* inoculation

Seed inoculation was done under shade in the field to reduce the bacterial cell death. Inoculated seeds were allowed to air-dry for a few minutes before planting. Two seeds were sown in each hole for both inoculated and uninoculated treatments. To avoid cross contamination, the uninoculated seeds were always planted first, followed by inoculated treatment. Soil ridges were made to separate inoculated and uninoculated treatments from each other in order to prevent cross contamination through rainwater movement. After sowing, the seeds were immediately covered with moist soil to avoid rhizobial cell death from desiccation. Planting was done using a spacing of 40 cm between rows and 10 cm between plants. Each experimental plot measured 2.4 m × 3.6 m (8.64 m^2^). Each year, field experiments were carried out during early August. Weeding was done manually by hoe at two weeks after seedling emergence, and three weeks later, if needed. To avoid cross contamination, weeding was done in the uninoculated plots first. Planting in 2013 was done in a field adjacent to the one used in 2012.

### Plant sampling and processing

At early pod-setting, five common bean plants were randomly dug up from each plot, placed in paper bags and transported to the laboratory, where each plant was separated into roots, nodules and shoots. The shoots were oven-dried at 70 °C for 48 h, weighed, and finely ground (0.85 mm sieve size) for ^15^N isotopic analysis. Non-legume plant species concurently sampled from inside the plots as done for legume were used as reference plants for determining soil mineral N uptake by bean plants and ^15^N/^14^N isotopic fractionation associated with N_2_ fixation. In total, eight and twelve reference plants were sampled and processed for ^15^N analysis in 2012 and 2013, respectively (Table 1).

**Table 1:** The δ^15^N values of reference plants used for estimating soil N uptake or %Ndfa by common bean at Galalicha during 2012 and 2013 cropping seasons

Plant species	δ^15^ N (‰)
2012	
*Ageratum conyzoides*	+4.65
*Amaranthus hybridus*	+4.67
*Datura stromanium*	+4.80
*Galinsonga parviflora cav*	+5.62
*Lepidium africanum*	+4.08
*Nicandra physalodes* (L.) Scop.	+5.69
*Raphonus raphanistrum*	+6.81
*Zea mays* L.	+5.83
2013	
*Achyranthes aspera*	+4.92
*Amaranthus palmeri*	+4.74
*Bidens pilosa*	+6.47
*Chenopodium ambrosioides*	+4.06
*Datura stramonium*	+5.47
*Erucastrum arabicum*	+6.95
*Lepidium africanum*	+4.96
*Leucus argentea*	+5.58
*Malva verticillata*	+6.19
*Parthenium hysterophorus*	+4.43
*Xanthium spinosum*	+4.36
*Zea mays* L.	+7.27

### ^15^N isotopic analysis and estimation of N-fixed and soil N uptake

Isotopic analysis was done at the University of Cape Town Stable Isotope Laboratory. About 2 to 3 mg finely ground shoot samples of bean and reference plants were weighed into aluminium capsules and fed onto a Thermo 2000 Elemental Analyzer coupled via a Thermo Conflo IV to a Thermo Delta V Plus stable light isotope mass spectrometer (Thermo, Corporation, Bremen, Germany). An internal standard of *Nasturtium spp.* was included in every five runs to correct for machine error during isotopic analysis in 2012, while Merck Gel (δ^15^N = 6.8‰) was included as standard after every twelve runs in 2013.

The isotopic composition of plant samples was calculated as (Mariotti et al. 1981):

$$ \updelta {}^{15}\mathrm{N}\left({\mbox{\fontencoding{U}\fontfamily{wasy}\selectfont\char104}} \right)=\frac{{\left({}^{15}\mathrm{N}/{}^{14}\mathrm{N}\right)}_{\mathrm{sample}}-\kern0.5em {\left({}^{15}\mathrm{N}/{}^{14}\mathrm{N}\right)}_{\mathrm{standard}}}{{\left({}^{15}\mathrm{N}/{}^{14}\mathrm{N}\right)}_{\mathrm{standard}}}\times 1000 $$

Where ^15^N/^14^N _sample_ and ^15^N/^14^N _standard_ are respectively the abundance ratio of the sample and air (the International Atomic Energy Agency standard).

The N content of each plant sample was determined as the product of N concentration (obtained directly from mass spectrometer) and shoot dry matter (Peoples et al. 2009).

The percent N derived from the fixation of atmospheric N_2_ (%Ndfa) by bean plants was calculated from the ^15^N abundance of the legume species and that of the non-fixing reference plant as (Shearer and Kohl 1986; Unkovich et al. 2008):

$$ \%\mathrm{Ndfa}=\frac{\delta^{15}{\mathrm{N}}_{\mathrm{ref}}-{\delta}^{15}{\mathrm{N}}_{\mathrm{leg}}}{\delta^{15}{\mathrm{N}}_{\mathrm{ref}}-\mathrm{B}}\times 100 $$

Where, δ^15^N_ref_ is the ^15^N natural abundance of non-fixing reference plant shoots used in the experiment, and δ^15^N_leg_ is the ^15^N natural abundance of common bean shoots. The B value is the ^15^N natural abundance of the legume (common bean) relying exclusively on N_2_ fixation for its N nutrition. The B value (−0.482‰) used to calculate %Ndfa in this study was obtained from Kimura et al. (2004). The combined mean δ^15^N value of the total number of reference plant species sampled from each experimental site was used in estimating %Ndfa of that site for the year concered.

The amount of N-fixed was calculated as (Maskey et al. 2001):

$$ \mathrm{N}-\mathrm{fixed}=\%\mathrm{Ndfa}\times \mathrm{legume}\  \mathrm{biomass}\;\mathrm{N} $$

Where legume biomass N was the N content of common bean shoots.

The soil N uptake was calculated by calculating the difference between total N and N-fixed.

### Grain yield

At physiological maturity, the total number of plants were counted from the inner three rows of each plot to obtain a stand count. At harvest, ten plants were randomly removed from three inner rows of each plot (2.4 m × 1.2 m = 2.88 m^2^). The pods were plucked, pooled, and grain removed from pods per plot, and weighed after air-drying to 11% moisture level as measured by a moisture meter (Model HOH-EXPRESS HE 50).

### Statistical analysis

The data collected were statistically analysed using the general linear model procedure of Statistical Analysis Software (SAS 9.0, Institute, Inc., Cary, NC, USA). Data on plant growth (measured as shoot dry matter), symbiotic parameters and grain yield were analysed using a three-way ANOVA in order to assess the treatment effect of bean varieties, inoculant types and phosphorus levels. Where treatments differed statistically, the Duncan’s multiple range test was used to separate the means at *p* ≤ 0.05. Correlation analysis was done using Pearson’s simple correlation coefficients to test the relationships between plant growth, symbiotic parameters and/or grain yield.

## Results

### Shoot δ^15^N of reference plants

The results of the shoot δ^15^N, whether minimum, maximum or combined mean of non-fixing reference plant species are presented in Table 1. In 2012, the δ^15^N of reference plants ranged from +4.08 to +6.81, and in 2013 from +4.06 to +7.27. The combined mean δ^15^N values of the reference plants used to estimate %Ndfa of bean were +5.30‰ and +5.45‰, respectively, in 2012 and 2013 (Table 2).

**Table 2:** The δ^15^N values of the reference plants sampled for estimating soil N uptake or %Ndfa by common bean

Year	No of plants	δ^15^ N values (‰)		
	sampled per site (n)	Minimum	Maximum	Mean ± SE
2012				
	8	4.08	6.81	5.30 ± 0.31
2013				
	12	4.06	7.27	5.45 ± 0.31

The values include the minimum, maximum and mean during 2012 and 2013 cropping seasons

### Effect of bean variety on plant growth, symbiotic performance and grain yield of common bean in 2012 and 2013

Plant growth measured (as shoot dry matter) was similar in 2012, but differed markedly between the two bean varieties in 2013 (Table 3). Shoot N concentration was similar for 2012 and 2013; N content was however higher in Hawassa Dume in 2013 but not 2012 (Table 3). Shoot δ^15^N values were also similar in 2012, but lower in Hawassa Dume than Ibbado in 2013. As a result, %Ndfa values were similar in the two bean varieties in 2012, but differed in 2013, with Hawassa Dume obtaining more N from fixation. Symbiotic N contribution was significantly higher in Hawassa Dume in 2012 and 2013, while soil N uptake was similar for both varieties during the two cropping seasons (Table 3). The Hawassa Dume variety produced significantly more grain yield than Ibbado in 2012 (1.84 vs. 1.65 t.ha^−1^) and 2013 (2.05 vs. 1.86 t.ha^−1^).

### Effect of *Rhizobium* inoculation on plant growth, symbiotic performance and grain yield of common bean in 2012 and 2013

In 2012, rhizobial inoculation increased shoot DM significantly over uninoculated plants, with strain HB-429 inducing greater plant growth than strain GT-9 (Table 3). A similar result was obtained in 2013. Although, *Rhizobium* inoculation had no effect on shoot N concentration in 2012 it increased it in 2013 relative to uninoculated control. N content was however higher in inoculated plants than uninoculated in 2012 despite having similar N levels (Table 3). Although bacterial inoculation showed no effect on shoot δ^15^N in 2012, it decreased it in 2013 relative to uninoculated control. As a result, percent N derived from fixation was similar in 2012, but higher in inoculated plants in 2013. However, the amounts of N-fixed differed in both 2012 and 2013, with the inoculated plants making a significantly greater N contribution than their uninoculated counterparts in both years. Soil N uptake by bean plants was greater with *Rhizobium* HB-429 inoculation in 2012, but similar for inoculated and uninoculated plants in 2013 (Table 3). Bean inoculation with the two rhizobial strains significantly increased grain yield (Table 3), as well as the number of pods per plant, number of seeds per pod, 100-seed weight and grain yield compared to uninoculated control (data not shown).

**Table 3:** Plant growth, symbiotic performance and grain yield of common bean varieties in response to rhizobial inoculation and P application at Galalicha during the 2012 and 2013 cropping seasons

Treatments	Shoot dry matter g plant^−1^	N concentration %	N content g plant^−1^	δ^15^ N ‰	Ndfa %	N-fixed kg.ha^−1^	Soil N uptake kg.ha^−1^	Grain yield (t.ha^−1^)
2012								
Variety								
Hawassa Dume	15.23 ± 0.43a	2.2 ± 0.04a	0.34 ± 0.01a	3.2 ± 0.04a	36 ± 0.98a	23 ± 1.38a	40 ± 1.65a	1.84 ± 0.11a
Ibbado	15.13 ± 0.34a	2.2 ± 0.04a	0.33 ± 0.01a	3.4 ± 0.06a	34 ± 1.18a	21 ± 1.10b	41 ± 1.36a	1.65 ± 0.09b
*Rhizobium*								
Uninoculated	14.03 ± 0.44c	2.2 ± 0.04a	0.30 ± 0.01c	3.5 ± 0.11a	33 ± 0.97a	18 ± 0.99c	38 ± 1.70b	1.34 ± 0.08b
HB-429	16.61 ± 0.47a	2.2 ± 0.07a	0.37 ± 0.02a	3.3 ± 0.04a	35 ± 1.83a	25 ± 2.13a	44 ± 2.19a	1.97 ± 0.13a
GT-9	14.89 ± 0.40b	2.2 ± 0.04a	0.32 ± 0.01b	3.1 ± 0.10a	36 ± 0.02a	21 ± 0.96b	39 ± 1.40b	1.93 ± 0.13a
Phosphorus								
0	12.57 ± 0.34c	2.0 ± 0.04c	0.25 ± 0.01d	3.5 ± 0.08a	32 ± 1.32b	15 ± 0.63b	32 ± 1.48c	0.98 ± 0.04c
10	13.95 ± 0.48b	2.0 ± 0.03c	0.28 ± 0.01c	3.4 ± 0.11a	33 ± 1.91b	17 ± 1.28b	35 ± 1.37c	1.72 ± 0.07b
20	16.85 ± 0.30a	2.5 ± 0.05a	0.42 ± 0.02a	3.4 ± 0.04a	35 ± 0.77ab	27 ± 0.96a	51 ± 1.65a	2.12 ± 0.16a
30	17.33 ± 1.34a	2.2 ± 0.05b	0.38 ± 0.03b	3.1 ± 0.10b	38 ± 1.68a	27 ± 2.03a	43 ± 1.39b	2.18 ± 0.12a
*F-statistic*								
Variety (V)	0.16^NS^	1.22^NS^	0.28^NS^	3.12^NS^	3.12^NS^	4.48^*^	0.97^NS^	6.32^*^
*Rhizobium* (R)	36.32^***^	1.47^NS^	28.72^***^	1.16^NS^	1.16^NS^	14.77^***^	10.34^***^	23.92^***^
Phosphorus (P)	42.41^***^	50.47^***^	126.30^***^	3.91^*^	3.91^*^	46.94^***^	57.77^***^	24.02^***^
V x R	0.05^NS^	6.52^**^	5.58^**^	0.91^NS^	0.92^NS^	3.88^*^	0.59^NS^	2.84^NS^
V x P	3.85^*^	1.23^NS^	5.83^**^	0.77^NS^	0.77^NS^	0.96^NS^	4.87^**^	1.34^NS^
R x P	2.76^*^	5.94^***^	3.97^**^	5.17^**^	5.17^**^	7.65^***^	1.34^NS^	3.09^*^
V x R x P	1.81^NS^	1.83^NS^	5.71^***^	0.90^NS^	0.90^NS^	1.26^NS^	4.02^***^	2.21^NS^
2013								
Variety								
Hawassa Dume	16.20 ± 0.46a	2.4 ± 0.06a	0.39 ± 0.02a	2.5 ± 0.10b	50 ± 1.62a	38 ± 2.41a	35 ± 1.46a	2.05 ± 0.12a
Ibbado	15.48 ± 0.39b	2.3 ± 0.05a	0.36 ± 0.01b	3.0 ± 0.11a	41 ± 1.85b	28 ± 2.01b	37 ± 1.24a	1.86 ± 0.10b
*Rhizobium*								
Uninoculated	14.36 ± 0.43c	2.2 ± 0.07b	0.32 ± 0.02b	3.2 ± 0.10a	38 ± 1.61b	23 ± 1.68b	37 ± 1.84a	1.72 ± 0.06b
HB-429	17.21 ± 0.50a	2.4 ± 0.07a	0.41 ± 0.02a	2.6 ± 0.15b	49 ± 2.53a	39 ± 3.11a	38 ± 1.68a	2.59 ± 0.10a
GT-9	15.95 ± 0.53b	2.4 ± 0.06a	0.39 ± 0.02a	2.5 ± 0.11b	50 ± 1.92a	37 ± 2.68a	35 ± 1.47a	2.56 ± 0.07a
Phosphorus								
0	12.91 ± 0.27c	2.2 ± 0.07c	0.28 ± 0.01c	3.3 ± 0.12a	36 ± 2.09b	19 ± 1.50c	33 ± 1.38c	0.96 ± 0.04c
10	14.13 ± 0.40b	2.2 ± 0.08bc	0.32 ± 0.02b	2.7 ± 0.19b	47 ± 3.13a	29 ± 2.78b	30 ± 1.62c	1.72 ± 0.06b
20	17.90 ± 0.30a	2.5 ± 0.07a	0.45 ± 0.02a	2.6 ± 0.12b	47 ± 1.96a	40 ± 2.71a	43 ± 1.75a	2.59 ± 0.10a
30	18.41 ± 0.47a	2.4 ± 0.07ab	0.45 ± 0.02a	2.4 ± 0.13b	51 ± 2.11a	44 ± 3.19a	39 ± 1.61b	2.56 ± 0.07a
*F-statistic*								
Variety (V)	10.63^**^	3.70^NS^	9.40^**^	25.96^***^	25.97^***^	27.65^***^	1.98^NS^	24.90^***^
*Rhizobium* (R)	53.56^***^	3.46^*^	28.72^***^	13.77^***^	13.77^***^	23.29^***^	1.12^NS^	32.09^***^
Phosphorus (P)	57.49^***^	5.69^*^	24.88^***^	21.07^***^	21.07^***^	35.49^***^	13.03^**^	202.95^***^
V x R	0.33^NS^	2.08^NS^	2.63^NS^	0.31^NS^	0.31^NS^	2.79^NS^	1.26^NS^	3.14^NS^
V x P	2.68^NS^	1.75^NS^	2.92^*^	2.08^NS^	2.08^NS^	0.90^NS^	3.76^*^	2.63^NS^
R x P	4.14^**^	1.83^NS^	1.32^NS^	2.04^NS^	2.04^NS^	2.00^NS^	2.97^*^	3.88^**^
V x R x P	1.07^NS^	0.44^NS^	0.77^NS^	0.96^NS^	0.96^NS^	1.17^NS^	0.56^NS^	2.63^NS^

Values (Mean ± SE) followed by dissimilar letters in a column are significantly different at *: p ≤ 0.05; **: *p* ≤ 0.01; ***: *p* ≤ 0.001; NS = non-significant

### Effect of phosphorus supply on plant growth, symbiotic performance and grain yield of common bean in 2012 and 2013

Applying P to bean plants increased plant growth, shoot N concentration and N content in both 2012 and 2013, with greater N accumulation at the higher P levels (Table 3). However, P supply decreased δ^15^N values relative to zero-P treatment in both 2012 and 2013 with the lowest δ^15^N being recorded at 30 kg P ha^−1^. As a result, percent N derived from fixation increased from 32% at zero-P to 38% at 30 kg P ha^−1^ in 2012 and 36% to 51% respectively in 2013. The amount of N-fixed also expectedly increased from 15 kg N ha^−1^ at zero-P to 38 kg N ha^−1^ at 30 kg P ha^−1^ in 2012, and 19 to 44 kg N ha^−1^ respectively in 2013 (Table 3). Soil N uptake and grain yield by bean plants also increased significantly with rising P supply.

### Variety x *Rhizobium* interaction

**Fig. 1:**
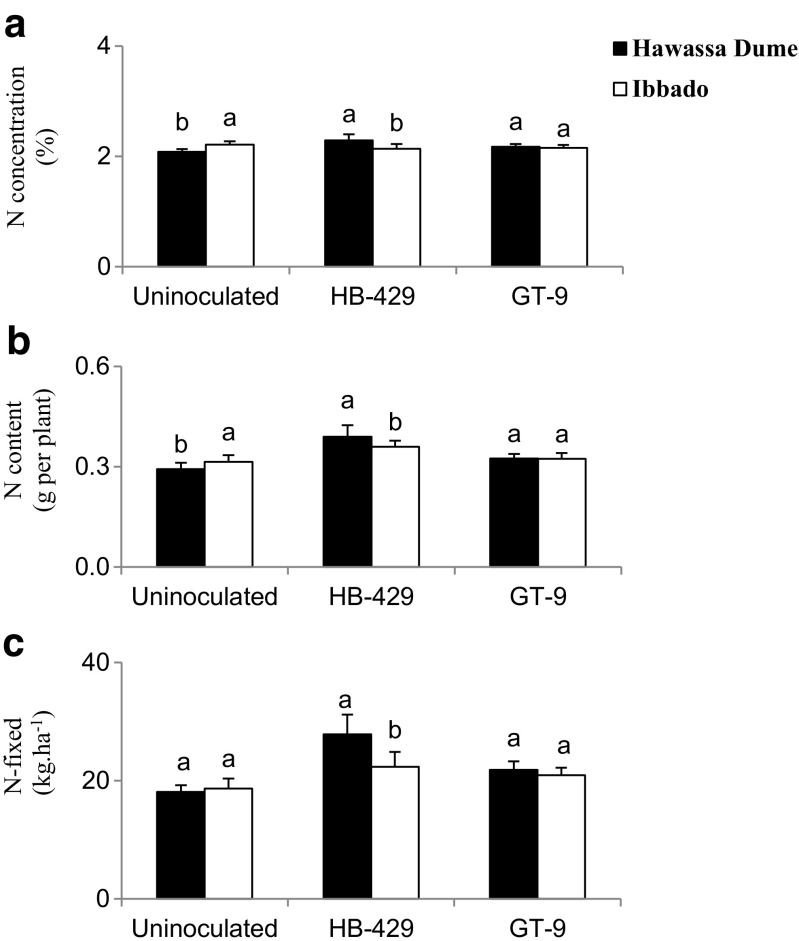
The interactive effect of variety x *Rhizobium* at Galalicha in 2012 on: **a** N concentration **b** N content; and **c** N-fixed. Vertical lines on bars represent S.E of the means

The variety x *Rhizobium* interaction was significant for N concentration, N content and N-fixed in 2012 (Table 3). The results showed a consistently greater N concentration, N content and amount of N-fixed in Hawassa Dume over Ibbado when inoculated with strain HB-429, in contrast to the much lower N concentration and N content under uninoculated conditions (Fig. 1a, b and c). However, Ibbado outperformed Hawassa Dume in uninoculated plots in terms of shoot N concentration, N content and N-fixed. In contrast, strain GT-9 produced similar results with Hawassa Dume and Ibbado as host plants (Fig. 1a, b and c).

### Variety x phosphorus interaction

**Fig. 2:**
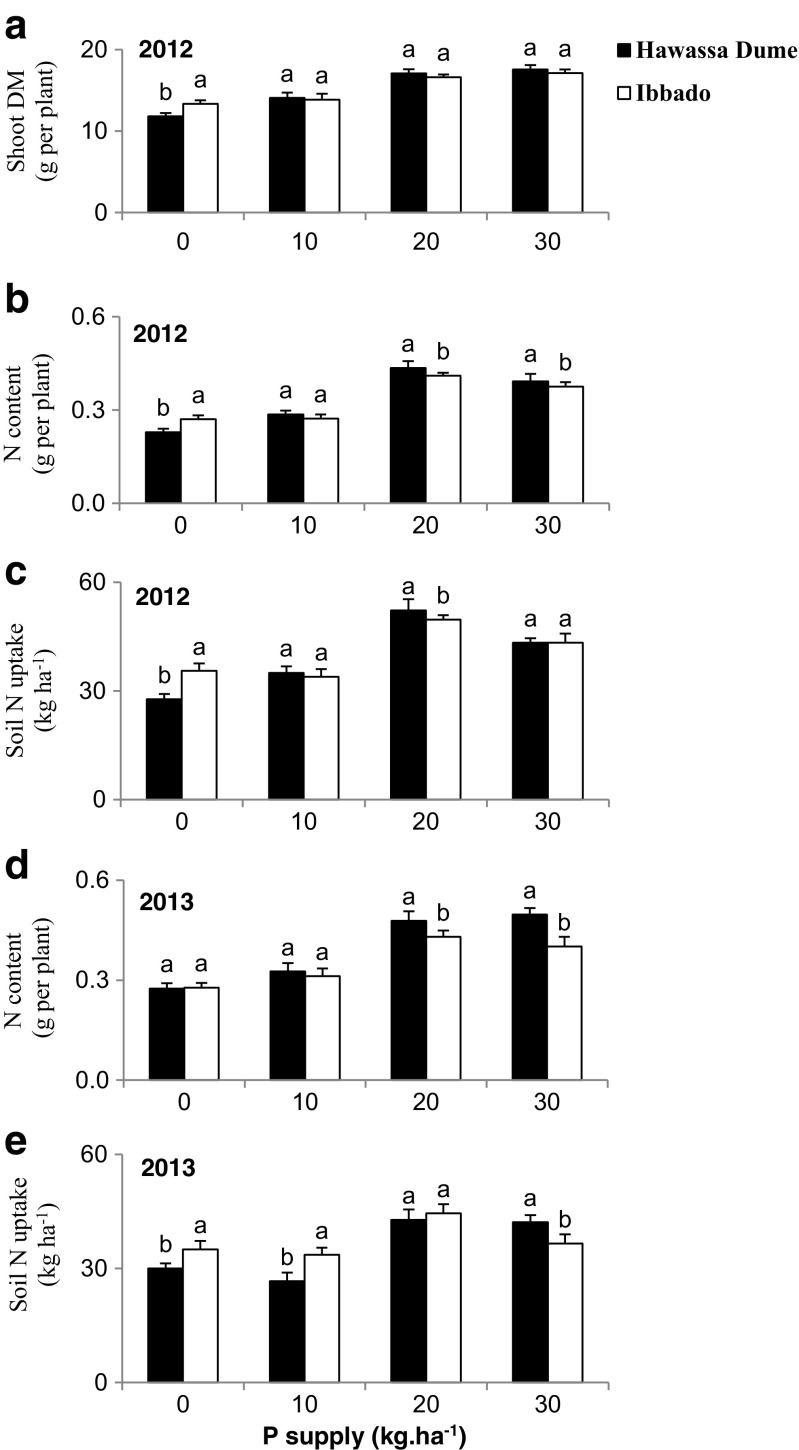
The interactive effect of variety x phosphorus at Galalicha on: **a** Shoot DM; **b** & **d** N content; and **c** & **e** Soil N uptake. Vertical lines on bars represent S.E of the means. The sowing year is indicated in each figure

The variety x phosphorus interaction was significant for shoot DM in 2012, as well as for N content and soil N uptake in both 2012 and 2013 cropping seasons (Table 3). Analysis of the results revealed similar amounts of shoot biomass at all P levels, except at zero-P in 2012 (Fig. 2a), greater shoot N content at 20 and 30 kg P ha^−1^ in Hawassa Dume than Ibbado in both 2012 and 2013 (Fig. 2b and d), but similar at 10 and much lower for Hawassa Dume at 0 kg P ha^−1^ (Fig. 2b). Soil N uptake was higher in Hawassa Dume over Ibbado at 20 kg P ha^−1^ in 2012 and at 30 kg P ha^−1^ in 2013, but lower than Ibbado in zero-P control plants in 2012 and 2013 (Fig. 2c and e).

### *Rhizobium* x phosphorus interaction

**Fig. 3:**
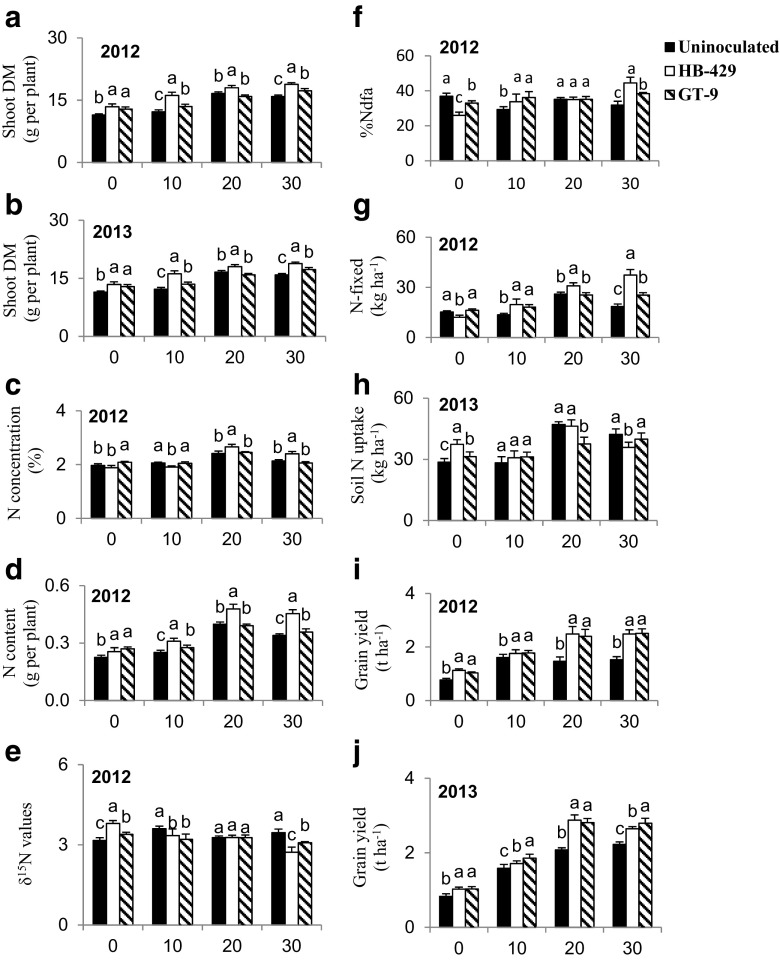
The interactive effect of *Rhizobium* x phosphorus on: **a** & **b** Shoot DM; **c** N concentration; **d** N content; **e** δ^15^N values; **f** %Ndfa; **g** N-fixed; **h** Soil N uptake; and **i** & **j** Grain yield. Vertical lines on bars represent S.E of the means. The sowing year is indicated in each figure

The *Rhizobium* x phosphorus interaction was significant for shoot DM, soil N uptake and grain yield in 2012 and 2013, as well as for N concentration, N content, δ^15^N, %Ndfa and N-fixed in 2012 (Table 3). Analysis of *Rhizobium* x phosphorus interaction revealed greater shoot biomass accumulation by inoculated bean plants over their uninoculated counterparts at 0, 10 and 30 kg P ha^−1^ treatments in 2012 and 2013 (Fig. 3a and b). At 20 kg P ha^−1^, the shoot biomass produced by uninoculated and strain GT-9 inoculated plants were similar in 2012 and 2013, but lower than that of strain HB-429. The results also showed higher shoot N concentration at 20 and 30 kg P ha^−1^ when inoculated with strain HB-429, but lower levels at 0 and 10 kg P ha^−1^ in 2012 (Fig. 3c). But shoot N content was increased by inoculating with strains HB-429 and GT-9 at 0, 10 and 30 kg P ha^−1^ (Fig. 3d). At 20 kg P ha^−1^ strain HB-429 still yielded more shoot N content than strain GT-9 and the uninoculated plants. Shoot δ^15^N values were generally lower with inoculation at 10 and 30 kg P ha^−1^ but greater at zero-P (Fig. 3e). As a result, %Ndfa values were higher with *Rhizobium* inoculation at 10 and 30 kg P ha^−1^, but lower at zero-P (Fig. 3f). Amount of N-fixed was greater with strain HB-429, followed by GT-9 when plants were fed 10, 20 and 30 kg P ha^−1^ (Fig. 3g). The N contribution by strain HB-429 was however much lower at zero-P. At zero-P, the inoculated plants took up more soil N than uninoculated, while at higher P levels, the uninoculated bean plants could take up the same or more soil N than strain HB-429 or GT-9 (Fig. 3h). In the two cropping seasons, *Rhizobium* inoculation alone significantly increased grain yield when compared to uninoculated plants at zero-P (Fig. 3i and j). In fact, inoculation with either strains resulted in markedly greater grain yield than uninoculated plants at 10, 20 and 30 kg P ha^−1^ in both 2012 and 2013 (Fig. 3i and j).

### Correlation analysis

The correlation coefficients for pair-wise comparison of the degree of association between and among plant growth, symbiotic performance and grain yield of common bean in 2012 and 2013 are presented in Table 4. The analyses revealed a positive correlation between shoot dry matter and symbiotic parameters (%Ndfa and N-fixed) in 2012 and 2013. Similarly, the results showed strong relationships between all parameters analysed, and %Ndfa of plants. N-fixed was positively correlated with soil N uptake in both cropping seasons. However, %Ndfa was negatively correlated with δ^15^N values and soil N uptake in both cropping seasons. But more importantly, N-fixed and %Ndfa both correlated positively with grain yield, indicating the importance of N_2_ fixation in legume grain yield.

**Table 4:** Correlation (r) among plant growth, symbiotic performance and grain yield of common bean varieties grown at Galalicha in Ethiopia, during 2012 and 2013 cropping seasons

Parameters	Significance			
	2012	*p value*	2013	*p value*
	r		r	
Shoot dry matter vs. %Ndfa	0.20	*	0.54	***
Shoot dry matter vs. N-fixed	0.73	***	0.84	***
Shoot dry matter vs. soil N uptake	0.80	***	0.50	***
%Ndfa vs. δ^15^N	−1.00	***	−1.00	***
%Ndfa vs. N-fixed	0.74	***	0.83	***
%Ndfa vs. soil N uptake	−0.17	NS	−0.24	*
%Ndfa vs. grain yield	0.29	**	0.50	***
N-fixed vs. δ^15^N	−0.74	***	−0.83	***
N-fixed vs. soil N uptake	0.51	***	0.27	***
N-fixed vs. grain yield	0.59	***	0.70	***

## Discussion

The ^15^N natural abundance technique was used to evaluate N contribution by common bean grown at Galalicha in Southern Ethiopia. The combined mean δ^15^N values of eight and twelve reference plants (+5.30 and +5.45‰ in 2012 and 2013, respectively) were markedly greater than the highest δ^15^N of common bean plants sampled (Table 2). This thus permitted estimation of %Ndfa and amount of N-fixed for common bean in Ethiopia as reported for grain legumes elsewhere (Nyemba and Dakora 2010; Belane et al. 2013; Mohale et al. 2014).

Of the two bean varieties evaluated in this study, Hawassa Dume showed better nodulation (data not shown) and greater plant growth compared to Ibbado (Table 3). Common bean has a high P-demand for nodulation and optimal growth (Silva et al. 2014), and some varieties can tolerate low P than others from better P-use efficiency (Vadez and Drevon 2001), which in turn can enhance N_2_ fixation in common bean (Vadez et al. 1999). Ethiopian soils are however categorized as P-deficient (Halm 1977), with extractable, plant-available P at the study site ranging from 0.65 mg.kg^−1^ in 2012 to 1.00 mg.kg^−1^ in 2013. The better plant growth and symbiotic performance by Hawassa Dume over Ibbado could be attributed to differences in the mechanisims used to take up P from the rhizosphere. In fact, shoot P concentrations were found to be higher in Hawassa Dume due to greater acid phosphatase activity in the rhizosphere and organs when compared to Ibbado (data not shown). The superior performance of both bean varieties in 2013 over 2012 could be due to improved soil P availability in 2013, as well higher rainfall (754.4 mm in the 2013 vs 746.6 mm in 2012) as well as increased soil N levels.

Hawassa Dume was symbiotically more effective than Ibbado, measured here as lower shoot δ^15^N values, greater %Ndfa and larger amounts of N-fixed (23–38 kg N ha^−1^ for Hawassa Dume vs. 20–29 kg N ha^−1^ for Ibbado). Relative to N contribution by other grain legumes (Belane et al. 2013; Mohale et al. 2014; Mokgehle et al. 2014), N contribution by common bean in this study is quite low. However, when compared to the 5–31 kg N ha^−1^ estimated for this legume elswhere in Africa (Ronner and Franke 2012), then the amount of N-fixed in this study is reasonably high. As a result of the known poor symbiotic performance of common bean (Kellman et al. 2005; Sánchez et al. 2014), soil N uptake by both varieties in this study was much higher than N-fixed (Table 3). There was thus a greater dependence on soil N for growth and grain yield by bean grown in Ethiopia. Generally, common bean has been recognized as a poor N_2_-fixer (Nyemba and Dakora 2010; Kabahuma 2013; Sánchez et al. 2014). That not withstanding, Hawassa Dume contributed much more symbiotic N and therefore, produced significantly greater grain yield when compared to Ibbado variety (Table 3).

Rhizobial inoculation is often done by farmers as an insurance against nodulation failure due to the presence of abundant ineffective native rhizobia in agricultural soils. In this study, both common bean varieties responded positively to inoculation with *Rhizobium* strains HB-429 and GT-9 under Ethiopia conditions (Table 3), a finding consistent with previous reports (Trabelsi et al. 2011; Kawaka et al. 2014). The significantly increased plant growth, symbiotic performance and grain yield of the test bean varieties from inoculation with *Rhizobium* strains HB-429 and GT-9 together provide direct evidence for the poor symbiotic competitiveness and effectiveness of native rhizobia nodulating bean in Ethiopian soils, while affirming the symbiotic superiority of the introduced strain. The increased plant growth as a result of *Rhizobium* inoculation also resulted in greater grain yield (Table 3). This was evidenced in this study by the significantly positive correlation between percent N derived from fixation and bean grain yield, as well as significantly marked correlation between amount of N-fixed and grain yield (Table 4). *Rhizobium*-inoculated common bean plants accumulated significant amounts of symbiotic N when compared to uninoculated controls in both 2012 and 2013. This improved N nutrition from enhanced N supply via N_2_ fixation by the introduced strains no doubt resulted in greater photosynthate production for higher grain yield. These results are consistent with the findings of Kawaka et al. (2014), which showed that bean inoculation with an effective strain increased N nutrition and grain yield. These findings are also in agreement with those of Elkoca et al. (2010) and Zafar-Allah et al. (2007), who reported a stimulatory effect of *Rhizobium* inoculation on growth and symbiotic performances of common bean.

In this study, the estimated amounts of N-fixed by *Rhizobium*-inoculated plants ranged from 20 to 31 kg N ha^−1^, levels within the range (20 to 60 kg N ha^−1^) reported for *Rhizobium-*inoculated common bean in Brazil (Da Silva et al. 1993). The amount of N-fixed with rhizobial inoculation of common bean in a study by Bambara and Ndakidemi (2010a, b) also ranged from 8.6 to 33 kg ha^−1^. Here, the application of P to bean resulted in significant increases in plant growth, symbiotic performance and grain yield (Table 3), a finding consistent with the results of P fertilisation of common bean (Mourice and Tryphone 2012; Namugwanya et al. 2014). In Ethiopia, common bean varieties responded positively to exogenous P supply at a moderate 20 and 30 kg ha^−1^. The similar response by bean plants to 20 or 30 kg P ha^−1^ support the recommended rate of 20 kg P ha^−1^ for common bean as optimal for plant growth and grain yield by the Extension Service (Turuko and Mohammed 2014). Also, the increased nodule number and nodule biomass (data not shown), as well as shoot dry matter (Table 3) with P supply to common bean, is consistent with the report by Pereira and Bliss (1987) and Olivera et al. (2007) for common bean.

The application of P to bean plants also resulted in decreased shoot δ^15^N values, an indication of higher N_2_ fixation (Table 3) and this agrees with the findings of Chagas et al. (2010). However, there was a positive synergistic effect of *Rhizobium* inoculation and P-fertilisation on plant growth and symbiotic performance, as indicated by the lower shoot δ^15^N values, greater %Ndfa, and increased amount of N-fixed in plants treated to P and *Rhizobium* (Table 3). Even with P supply alone, there was a marked growth and symbiotic response in this study, thus indicating the need for P fertilisation of these P-deficient soils of Ethiopia before planting beans. The performance of correlation analysis found a strong and significant relationship between plant growth and symbiotic parameters, a result which supports the existence of a functional link between plant growth and symbiotic functioning in common bean (Belane and Dakora 2010; Nkot et al. 2013; Qureshi et al. 2013; Mohale et al. 2014). It is clear from those studies, as well as this one, that N_2_ fixation is important for plant growth and grain yield of nodulated food legumes, and is a cheap substitute for the use of N fertilizers.

Taken together, the results obtained in this study have shown that rhizobial inoculation and P application (20 kg P ha^−1^) can improve plant growth, symbiotic performance and grain yield of common bean varieties grown in the low-P soils of Ethiopia. The greater plant growth and enhanced N nutrition in *Rhizobium*-inoculated, P-fed plants translated into increased grain yield, an agronomic practice that could boost bean production in Ethiopia. The significant interactive effects of variety x *Rhizobium*, variety x phosphorus and *Rhizobium* x phosphorus further support the view that supplying these inputs to Ethiopian farmers can increase bean grain yield for food and nutritional security.
